# Left Main Coronary Artery Disease: The Forgotten Lead of Electrocardiogram Is Predictive

**DOI:** 10.7759/cureus.28391

**Published:** 2022-08-25

**Authors:** Pradnya Brijmohan Bhattad, Akil A Sherif, Ajay K Mishra, Mazen Roumia

**Affiliations:** 1 Cardiovascular Medicine, Saint Vincent Hospital, University of Massachusetts (UMass) Chan Medical School, Worcester, USA

**Keywords:** proximal left anterior descending artery disease, avr lead, ecg, left main coronary artery disease (lmcad), subendocardial ischemia

## Abstract

An ST segment depression in eight or more leads along with ST segment elevation in lead aVR or V1, especially occurring during ischemic symptoms, has a very high predictive accuracy of left main or three-vessel disease, or tight proximal left anterior descending (LAD) coronary artery stenosis. We describe a classic case of a patient who presented with ST elevation in the lead aVR with diffuse ST segment depression during anginal symptoms and was found to have severe disease in the distal left main, ostial circumflex, and left anterior descending artery on an emergent coronary angiogram.

## Introduction

An ST segment elevation in lead aVR especially during anginal symptoms should have a very low threshold for emergent angiographic evaluation given the high predictivity for significant left main disease or multivessel coronary artery disease. In a meta-analysis of 27 articles, ST elevation of >0.05 mv and the degree of ST elevation in aVR were reported to be independent predictors of left main disease and myocardial infarction in non-ST elevation acute coronary syndrome (ACS) [1].

The aVR ST segment elevation has been reported in the literature to signify proximal left anterior descending artery (LAD) obstruction proximal to the first major septal branch [2]. These reports led Yamaji et al. to investigate aVR ST segment elevation in patients with left main coronary artery (LMCA) obstruction. They studied 16 patients who presented within 12 hours of an acute coronary syndrome (ACS) that were subsequently, angiographically proven to have left main disease as the culprit lesion. In this group, 14 out of 16 (88%) patients were found to have aVR ST elevation of >0.05 mV [3]. 

## Case presentation

A 44-year-old male with a history of spina bifida, hypertension, dyslipidemia, and ongoing tobacco use presented with sudden onset of pressure-like substernal chest pain radiating down both arms with associated diaphoresis and dyspnea. He was hemodynamically stable at the time of presentation. He did not have any palpitations, dizziness, lightheadedness, presyncope, syncope, orthopnea, paroxysmal nocturnal dyspnea, or peripheral edema. He did not have any previous similar episodes of chest pain in the past. He was not very active at baseline due to his spina bifida that involves deformities in his bilateral lower extremities but he was able to ambulate with the assistance of crutches for the past several years.

**Figure 1:**
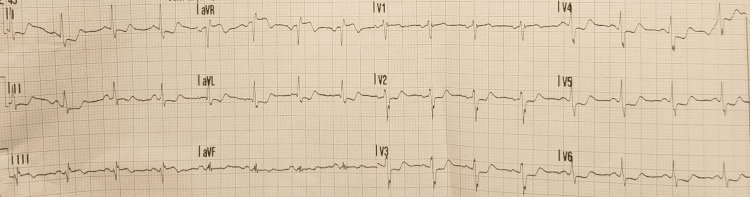
Electrocardiogram (ECG) shows diffuse ST segment depression along with ST segment elevation in lead aVR. This ECG was obtained while the patient actively complained of typical anginal symptoms and is suggestive of extensive sub-endocardial ischemia.

The patient called emergency medical services and was immediately brought to the emergency room for further evaluation. An electrocardiogram (ECG) was obtained in the ambulance when the patient actively complained of severe substernal chest pain while being transported to the emergency room (Figure 1). His heart rate was 90s/min, blood pressure 140s/70s mmHg, and oxygen saturation of 98% on room air with no rales or murmurs on physical examination at presentation.

The patient received a full dose of aspirin 325 mg by mouth while being transported to the emergency room in the ambulance. In the emergency room, he received a heparin bolus and his chest pain was managed with as-needed intravenous morphine and sublingual nitroglycerin. Given his presentation, he was emergently taken for cardiac catheterization. An emergent coronary angiogram (Videos 1-6) showed severe stenosis in the distal left main, severe stenosis in the ostial and mid-segment of the LAD, and the ostial segment of the left circumflex artery (LCx). The right coronary artery was angiographically normal. 

**Video 1:** Coronary angiogram showing severe disease in mid LAD LAD:  Left anterior descending artery

**Video 2:** Coronary angiogram showing severe disease in the distal left main, ostial LAD, mid LAD, and ostial LCx vessels LAD: Left anterior descending artery, LCx: Left circumflex artery

**Video 3:** Coronary angiogram showing severe disease in the distal segment of the left main and mid-segment of LAD LAD: Left anterior descending artery

**Video 4:** Severe disease in the mid LAD and distal left main well visualized on the coronary angiogram LAD: Left anterior descending artery

**Video 5:** Severe disease in ostial LAD, ostial LCx, and distal left main demonstrated on coronary angiogram LAD: Left anterior descending artery, LCx: Left circumflex artery

**Video 6:** Coronary angiogram showing severe disease in the distal left main, ostial LAD, ostial LCx, and mid LAD LAD: Left anterior descending artery, LCx: Left circumflex artery

An intra-aortic balloon pump was placed after the coronary angiogram for circulatory support. Cardiothoracic surgery was consulted for further evaluation of severe disease in the distal left main, ostial LAD, and ostial LCx vessels. The patient eventually underwent two-vessel coronary artery bypass grafting during the same hospitalization for multivessel coronary disease with significant left main disease. He performed well after surgical revascularization with complete symptom relief and remained hemodynamically stable. He was eventually discharged home with cardiac rehabilitation on optimal guideline-directed medical management. A transthoracic echocardiogram revealed left ventricular ejection fraction was 50% to 55% with no regional wall motion abnormalities before discharge. He will be followed as an outpatient. 

## Discussion

Kosuge et al. retrospectively studied 310 patients with ACS. In multivariate analysis, > 0.5 mm of ST elevation in aVR was noted to be the strongest predictor of left main coronary artery (LMCA) disease and three-vessel coronary artery disease with an odds ratio of 19.7 (p-<0.001). This pattern of ST elevation in aVR had a 86% specificity and a 95% negative predictive value in identifying LMCA disease [4].

Most studies suggest that ST segment elevations in the aVR lead can suggest LMCA involvement or proximal LAD obstructive lesions which can be clinically significant as well. Apart from ST elevation in aVR contributing to the identification of vascular territory, involvement of aVR too can portend to poor clinical prognosis. In a study of 333 ACS patients, aVR elevation and elevated troponin were predictors of myocardial infarction, the need for urgent revascularization, and death within 90 days [5]. Another study including 1042 patients with non-ST elevation ACS reported that ST depression and ST elevation in lead aVR in combination were associated with increased in-hospital cardiovascular mortality at one-year follow-up [6]. The ST elevation in lead aVR with diffuse ST depression is predictive of significant subendocardial ischemia [7].

## Conclusions

A careful systematic approach should be utilized when evaluating an electrocardiogram and integrating it with the clinical scenario. An ST-segment elevation in lead aVR during anginal symptoms must be emergently evaluated further for significant left main or multivessel coronary artery disease as these ECG findings have very high predictivity for significant subendocardial ischemia. 
